# Profiling the neutralizing antibody response in chronically HIV-1 CRF07_BC-infected intravenous drug users naïve to antiretroviral therapy

**DOI:** 10.1038/srep46308

**Published:** 2017-04-07

**Authors:** Xintao Hu, Yuanyuan Hu, Chunhong Zhao, Hongmei Gao, Kelli M. Greene, Li Ren, Liying Ma, Yuhua Ruan, Marcella Sarzotti-Kelsoe, David C. Montefiori, Kunxue Hong, Yiming Shao

**Affiliations:** 1 State Key Laboratory of Infectious Disease Prevention and Control, National Center for AIDS/STD Control and Prevention, Chinese Center for Disease Control and Prevention, Collaborative Innovation Center for Diagnosis and Treatment of Infectious Diseases, Beijing 102206, PR China; 2 Departments of Experimental Surgery, Duke University Medical Center, Durham, North Carolina 27710, USA; 3 Immunology, Duke University Medical Center, Durham, North Carolina 27710, USA

## Abstract

Characterizing neutralizing antibody (NAb) responses in individuals infected with diverse HIV-1 strains is necessary to reveal the novel targets for regional preventive and therapeutic strategies development. We evaluated the prevalence, breadth, and potency of NAb responses in 98 CRF07_BC-infected individuals using a large, multi-subtype panel of 30 tier 2-3 Env-pseudotyped viruses. Furthermore, we compared the neutralization pattern of CRF07_BC-infected people with that of subtype B’-infected individuals in China. Of the 98 plasma samples tested, 18% neutralized more than 80% of viruses in the panel, and 53% neutralized more than 50%, suggesting the presence of broadly NAbs in these individuals. A preferential intra-subtype neutralization of CRF07_BC was found. Notably, CRF07_BC-infected individuals generated higher neutralization titers against intra-subtype viruses than subtype B’-infected individuals with longer infection length. However, subtype B’-infected individuals mounted broader neutralization responses against inter-subtype viruses than CRF07_BC infection with shorter infection time, indicating the transition from narrow autologous to broad heterologous neutralization over time. Neutralization activity of the top six plasmas from each cohort was attributable to IgG fraction, and half of them developed CD4 binding site antibody reactivity. Heatmap analysis identified three statistically robust clusters of plasmas that offer valuable resources for further in-depth virological and immunological study.

Although highly active antiretroviral therapy suppresses HIV-1 replication effectively^1^, it does not fully eradicate the virus, produces undesirable side effects, requires life-long treatment to maintain suppression^2^, and is not accessible to all who need it. In contrast, preventive approaches, such as vaccination, provide more effective and economical protection against infectious diseases^3^. To date, successful vaccines against infectious diseases, such as influenza, hepatitis B, and measles provide protection through elicitation of protective neutralizing antibody (NAb) responses^4^,^5^,^6^. However, unlike these and many other viruses, HIV-1 has a high level of genetic variation, particularly in its envelope glycoprotein (Env), which is the sole target to induce the NAb response^7^. Additionally, HIV has evolved multiple mechanisms to evade the NAbs^8^. These features of HIV pose a tremendous challenge for vaccine development, particularly in the induction of broadly neutralizing antibodies (bNAbs) through conventional immunization^9^.

The lack of the detailed understanding of the immune responses induced by natural infection with HIV-1 might account for the limited success at eliciting effective NAb responses through vaccination. Natural infection provides an excellent opportunity to analyze and profile the immune response pattern mounted over the course of infection^10^ and may provide useful insights for rational immunogen design to induce similar immune responses and even lead to alternative biomedical prevention and therapy strategy development^11^,^12^,^13^,^14^. Therefore, it is essential to characterize NAb responses in individuals infected with diverse HIV-1 strains during HIV-1 infection. Our prior study on NAb response patterns in HIV-1 subtype B’ infection from a former plasma donor (FPD) cohort infected more than ten years found that around 29% of subjects mount broadly cross-reactive NAb responses^15^.

Previous molecular epidemiology studies from our laboratory^16^,^17^ and other researchers^18^ suggested that subtype B’ from Thailand and subtype C from India mixed in southwestern China-Yunan to form the 07_BC recombinant circulating subtype (CRF07_BC) and spread to Sichuan and Xinjiang of Western China by the drug trafficking route^16^,^17^. Initially, CRF07_BC mainly circulated in the intravenous drug users (IDUs) population of Western China and was further transmitted to Taiwan, Marco, and Japan, which made CRF07_BC become the main subtype in eastern Asia^19^. CRF07_BC was also called China C since it has the subtype C characteristics in the envelope protein portion^20^,^21^,^22^. The studies as mentioned above from mainland China^16^,^17^,^21^,^22^ together with multiple geographically-derived studies^19^,^23^ demonstrated the CRF07_BC likely originated from a common ancestor (a single or few founder virus) virus since the sequences could form a unique, homogeneous monophyletic cluster in the phylogenetic tree. The latest large-scale molecular epidemiology survey^24^ indicates HIV-1 CRF07_BC has become the dominant circulating strain for the IDU population in China and other countries in East Asia.

Our laboratory previously studied the biological and virological characteristics^25^ and cytotoxic T lymphocyte (CTL) response pattern^26^ of CRF07_BC infection. However, the humoral response pattern during infection caused by this unique recombinant subtype has not yet been adequately investigated. In the current study, we assessed the prevalence, breadth, and potency of NAb responses in CRF07_BC chronically infected individuals (infection time of 3–5 years) using a large multi-subtype panel of 30 tier 2–3 HIV-1 Env-pseudotyped virus strains which covered the main subtypes circulating in China and East Asia. We also comprehensively compared the neutralization pattern of this cohort with that of subtype B’ chronically infected individuals from the FPD cohort in China, infected for more than ten years, as described previously^15^,^27^. This comparison study in NAb patterns between the two cohorts offers a unique opportunity to observe and profile how broad NAbs evolve over time at the population level and in a single elite neutralizer, which could guide regional NAb-based prophylactic and therapeutic strategies development.

## Results

### NAb profiles of chronically CRF07_BC infected individuals

We list demographic and epidemiological data from this study cohort in Table S1. Plasma samples were tested using a standardized TZM-bl NAb assay against a panel of two tier 1 and 30 tier 2–3 Env-pseudotyped viruses (Table 1). All 98 plasma samples neutralized the two tier 1 strains, with geometric mean ID_50_ (50% inhibitory dose) titers (GMTs) against MW965.26 and SF162.LS being 34183 and 3903, respectively, which is in agreement with the higher neutralization sensitivity of the viruses^28^,^29^. We excluded the two tier 1 strains from further analysis as they were of little-to-no predictive value.

Neutralization breadth was defined as the fraction of strains neutralized at a detectable titer (ID_50_ > 20) for the remaining 30 tier 2–3 strains. We found that 18% of plasma samples (n = 18) neutralized ≥ 80% of viruses tested, 53% of plasma samples (n = 52) neutralized > 50% of viruses tested and that 1% (n = 1) neutralized the entire panel of viruses strains tested, indicating a relatively high prevalence of broadly cross-reactive NAb activity among this study population (Supplementary Fig. S1), which shared similar findings with other cohorts^30^,^31^. Based on neutralization breadth, the samples were divided into three groups (Table 1). Those with ≥80% or <50% neutralization breadth were defined as broadly cross-reactive neutralization group (BCN, n = 18) or non-broadly cross-reactive neutralization group (Non-BCN, n = 46), respectively. Samples with neutralization breadth between 50–80% were intermediate broadly cross-reactive neutralization group (M-BCN, n = 34). The classification criteria were the same as those used for the FPD cohort^15^.

Of the virus strains tested, CRF07_BC exhibited the highest neutralization sensitivities, with subtype-specific GMTs of 314.9 (BCN), 112.3 (M-BCN), 40.5 (Non-BCN), and 84.1 (overall study population), which are consistent with the cohort’s history of infection^16^,^18^. Among the ten subtype-matched viruses, the CH120.6 was the most resistant strain (Table 1) which was in line with other published study^29^. In contrast, CRF01_AE viruses exhibited the lowest sensitivities, with subtype-specific GMTs of 36.2 (BCN), 15.9 (M-BCN), 10.5 (Non-BCN), and 15.2 (overall study population). The order of subtype-specific GMTs of five subtypes tested was CRF07_BC > subtype C > subtype B > subtype A > CRF01_AE in all study groups except for the BCN group (subtype B < subtype A). Among all distinct strains, CH119.10 (CRF07_BC, tier 2 strain) exhibited the highest GMT value in all study groups except for the BCN group (CH181.12 > CH119.10), whereas SC422661.8 (subtype B, tier 2 strain) showed the lowest GMT in all study groups (Table 1).

The BCN group, which displayed the broadest neutralization responses, tended to have higher neutralization titers than M-BCN and Non-BCN groups that showed intermediate or low-level breadth. There was a strong significant positive correlation between neutralization breadth and potency across all 98 samples (R = 0.9664, p < 0.0001) (Supplementary Fig. S2a), as well as within the BCN (R = 0.6950, p = 0.0014) (Supplementary Fig. S2b), M-BCN (R = 0.6751, p < 0.0001) (Supplementary Fig. S2c), and Non-BCN (R = 0.9051, p < 0.0001) (Supplementary Fig. S2d) groups. These data indicated that the neutralization breadth and the potency might develop concurrently, a pattern analogous to that of the FPD cohort^15^ and other multiple geographically-derived cohorts^31^,^32^,^33^.

### Preferential intra-subtype neutralization of CRF07_BC-infected IDUs

**Figure 1:**
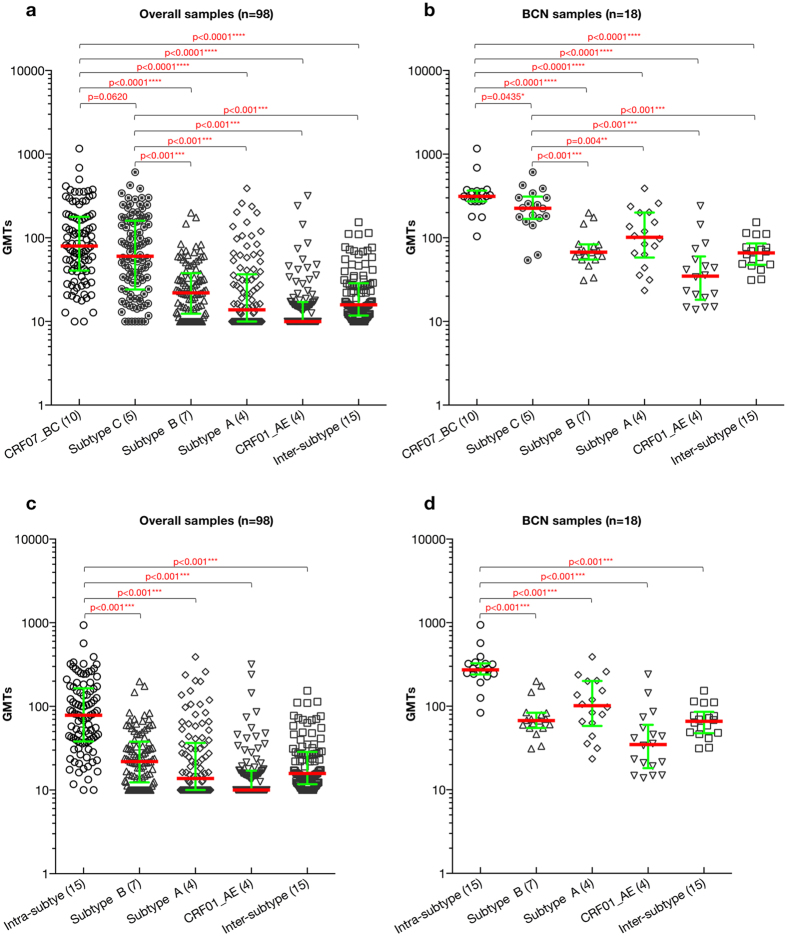
Comparison of GMTs between intra-subtype and subtype B/subtype A/CRF01_AE/inter- subtype virus strain set. The difference of GMTs between CRF-matched (CRF07_BC) and subtype C, subtype B, subtype A, CRF01_AE, inter-subtype specific (excluding all the CRF07_BC and subtype C viruses) virus sets is illustrated in the overall sample (**a**) and BCN sample population (**b**), respectively. While the difference of GMTs between intra-subtype (CRF07_BC and subtype C) and subtype B, subtype A, CRF01_AE, inter-subtype specific (excluding all the CRF07_BC and subtype C viruses) virus sets is exhibited in the overall sample (**c**) and BCN sample population (**d**), respectively. The number in the bracket following the particular subtype on the x-axis indicates the numbers of viruses used for comparison. P-values (two-tailed) are based on the Mann-Whitney U test. The error bars show the median with the interquartile range. The significant difference between the six groups is indicated: *0.01 < P < 0.05; **0.001 < P < 0.01; ***P < 0.001; ****P < 0.0001 (Mann-Whitney U test).

The NAb magnitude between subtype-matched CRF07_BC viruses and non-subtype-matched viruses, including subtype B, subtype A, CRF01_AE, and inter-subtype (excluding all the CRF07_BC and subtype C viruses as a whole), significantly differed (Fig. 1a, p < 0.0001 for all pairs). GMTs between subtype C and CRF07_BC viruses were comparable, which is in agreement with CRF07_BC envelope being derived from subtype C and its epidemiology history^16^,^18^. Within the BCN group, a significant difference in GMTs between subtype-matched CRF07_BC viruses and non-subtype-matched viruses was observed (Fig. 1b, p < 0.0001 for all pairs except for subtype C, p = 0.0435). Even the magnitude between subtype C viruses and non-subtype-matched viruses including subtype B, subtype A, CRF01_AE, and inter-subtype also significantly differed in both overall (Fig. 1a, p < 0.001 for all pairs) and BCN (Fig. 1b, p < 0.01 for all pairs) groups.

Combination of subtype-matched CRF07_BC (n = 10) and C (n = 5) as intra-subtype virus set (n = 15) was used to compare with inter-subtype virus sets. The significant statistic difference for the GMTs between intra-subtype viruses and non-subtype-matched viruses, including subtype B, subtype A, CRF01_AE, and inter-subtype was still held in both overall (Fig. 1c, p < 0.001 for all pairs) and BCN (Fig. 1d, p < 0.001 for all pairs) groups. Taken together, the data indicated the preferential intra-subtype neutralization in CRF07_BC infected IDU cohort as found in our FPD cohort^15^.

When investigating the association between neutralization and clinical parameters, we found that subtype-matched neutralization potency of CRF07_BC was positively correlated with viral load (Supplementary Fig. S3a, R = 0.2016, p = 0.0465) and negatively correlated with CD4 counts (Supplementary Fig. S3b, R = −0.2281, p = 0.0239) in the overall study population. Although we observed a similar tendency between the neutralization breadth against the subtype-matched virus of CRF07_BC and viral load (Supplementary Fig. S3c, R = 0.1756, p = 0.0836) or CD4 counts (Supplementary Fig. S3d, R = −0.1945, p = 0.055) as potency, neither achieved statistical significance. In contrast, both subtype-matched neutralization potency (R = 0.2815, p = 0.0040) and breadth (R = 0.2322, p = 0.0183) of subtype B’ was positively correlated with viral load in the overall FPD study population with longer infection time. This observation indicated that neutralization responses during earlier infection stage might be dominantly driven by the subtype-matched virus, targeting autologous antigens.

### IDU cohort with a shorter infection length generated higher neutralization titers against intra-subtype viruses than FPD cohort

We observed a significant difference in neutralization magnitude between the CRF07_BC infected IDU cohort and the subtype B’ infected FPD cohort (Supplementary Fig. S4a, p = 0.0065), indicating that individuals in IDU cohort mounted more robust NAb response than the FPD cohort (Supplementary Fig. S4a).

**Figure 2:**
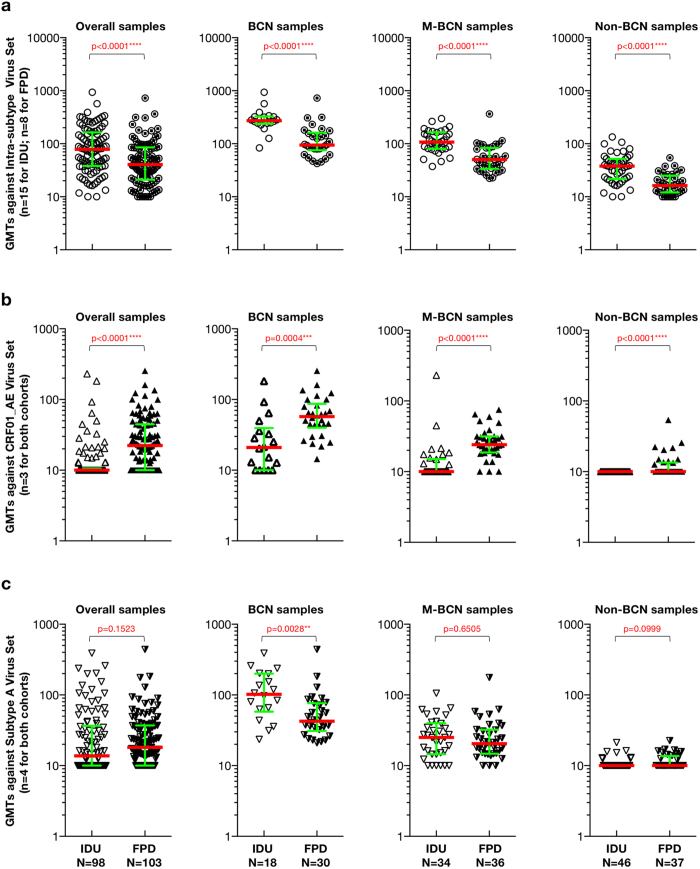
Comparison of NAb potency against the intra-subtype virus (a), CRF01_AE virus (b) and subtype A virus (c) between FPD and IDU cohort. The difference of GMTs of intra-subtype (**a**), CRF01_AE (**b**) and subtype A (c) virus set between IDU and FPD cohort is displayed for the different sample population. P-values (two-tailed) are based on the Mann-Whitney U test. The error bars show the median with the interquartile range. The significant difference between each group is indicated: *0.01 < P < 0.05; **0.001 < P < 0.01; ***P < 0.001; ****P < 0.0001 (Mann-Whitney U test).

To determine which portion of NAb magnitude contributed to these differences, we compared neutralization magnitude among each sample groups from both cohorts against intra-subtype viruses (Fig. 2a, the subtype B viruses used for FPD), and non-subtype-matched CRF01_AE (Fig. 2b) and subtype A (Fig. 2c) viruses. Since no subtype-matched (subtype B’) viruses were used for FPD cohort, it’s reasonable to combine the subtype-matched CRF07_BC and subtype C viruses as the intra-subtype virus set (n = 15) for IDU cohort to avoid the overestimate by only using CRF07_BC viruses closely genetically related to the strains circulating in IDU cohort. We observed significant differences between GMTs against intra-subtype viruses among all sample groups, including overall samples, BCN samples, M-BCN samples, and Non-BCN samples (Fig. 2a, p < 0.0001 for all pairs). All sample groups had higher GMTs in the IDU cohort than in the FPD cohort against their intra-subtype viruses (Fig. 2a, median GMTs: 78.31 versus 40.45 in the overall group; 272.31 versus 94.61 in the BCN group; 107.46 versus 50.45 in the M-BCN group; and 37.97 versus 16.34 in the Non-BCN group). Even the intra-subtype virus set for IDU cohort was restricted to the subtype C (n = 5) virus only, the significant statistic difference of NAb magnitude between both cohorts against the intra-subtype virus was still found (p ≤ 0.0186 for all pairs) among all samples group.

We found a significant difference in GMTs against CRF01_AE viruses (only matched-virus panel for both cohorts-Table S4) between IDU and FPD cohorts among overall samples (Fig. 2b, p < 0.0001), BCN samples (Fig. 2b, p = 0.0004), M-BCN samples (Fig. 2b, p < 0.0001), and Non-BCN samples (Fig. 2b, p < 0.0001). All sample groups had higher GMTs against CRF01_AE viruses (median GMTs: 22.33 versus 10.00 in overall sample; 57.22 versus 20.93 in BCN group; 24.04 versus 10.00 in M-BCN group; 10.00 versus 10.00 in Non-BCN group) in FPD cohort than the IDU cohort, demonstrating that participants from FPD cohort neutralized the CRF01_AE strains more efficiently than the patients in the IDU cohort. The only difference found in GMTs between IDU and FPD cohorts against subtype A virus set was in the BCN group (Fig. 2c, median GMTs: 101.76 versus 42.49 in BCN group) indicating, overall, that IDU cohort had equivalent neutralization activity to FPD cohort for subtype A virus set (Fig. 2c).

Collectively, these data demonstrated that IDU cohort with a shorter infection length generated stronger NAb responses targeting intra-subtype strains than FPD cohort with longer infection length.

### The FPD cohort with a longer infection length exhibited broader NAb activity against inter-subtype viruses than the IDU cohort

**Figure 3:**
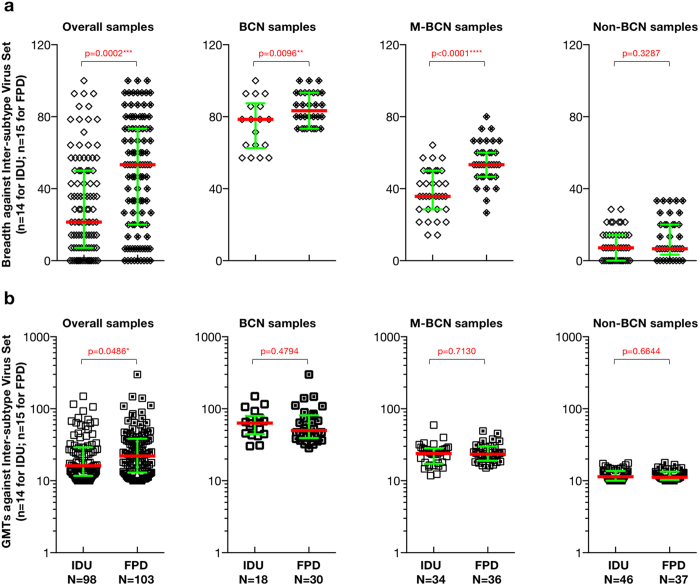
Comparison of NAb breadth and magnitude against inter-subtype virus set between FPD and IDU cohort. The difference of NAb breadth (**a**) and magnitude (**b**) against inter-subtype virus set between IDU and FPD cohort is illustrated for the different sample population. P-values (two-tailed) are based on the Mann-Whitney U test. The error bars show the median with the interquartile range. Different sample population including overall samples, BCN samples, M-BCN samples and Non-BCN samples, were labeled on the top of each figure, respectively. The Significant difference between each group is indicated: *0.01 < P < 0.05; **0.001 < P < 0.01; ***P < 0.001; ****P < 0.0001 (Mann-Whitney U test).

No significant difference between two cohorts for neutralization breadth against the overall virus set was found (Figure S4b). However, there was a significant difference in neutralization breadth against inter-subtype viruses (only matched-virus panel for both cohorts, Table S4) between IDU and FPD cohorts among overall group (Fig. 3a, p = 0.0002), BCN group (p = 0.0096), and M-BCN group (p < 0.0001) group. All sample groups except for the Non-BCN group developed broader cross-subtype NAb breadth in FPD cohort compared to IDU cohort against inter-subtype viruses (median breadth (%): 53.33 versus 21.43 in overall samples; 83.34 versus 78.57 in BCN samples; 53.33 versus 35.71 in M-BCN samples; 6.67 versus 7.14 in Non-BCN samples), indicating that infection length might be one of the critical factors for NAb breadth development. Regarding the prevalence of broadly and cross-reactive neutralizing antibody responses, the FPD population with longer infection length display a higher prevalence rate (29% versus 18% for FPD versus IDU), implying that NAb breadth became broader over time. We also found higher GMTs for FPD cohort than that of IDU cohort against inter-subtype viruses (Fig. 3b), suggesting more robust cross-reactive NAb response over time as well.

### CD4bs-directed binding specificity and isotype in top elite neutralizers

The top six elite neutralizers from each cohort were identified based on extensive neutralization screening (Table S2). Total IgG from these 12 samples was purified and quantified using a quantitative ELISA (Table S3). We evaluated the association between plasma and IgG neutralization against a subset of Env-pseudotyped viruses and demonstrated that plasma neutralization activity is ascribed primarily to the IgG fraction (Table 2).

**Figure 4:**
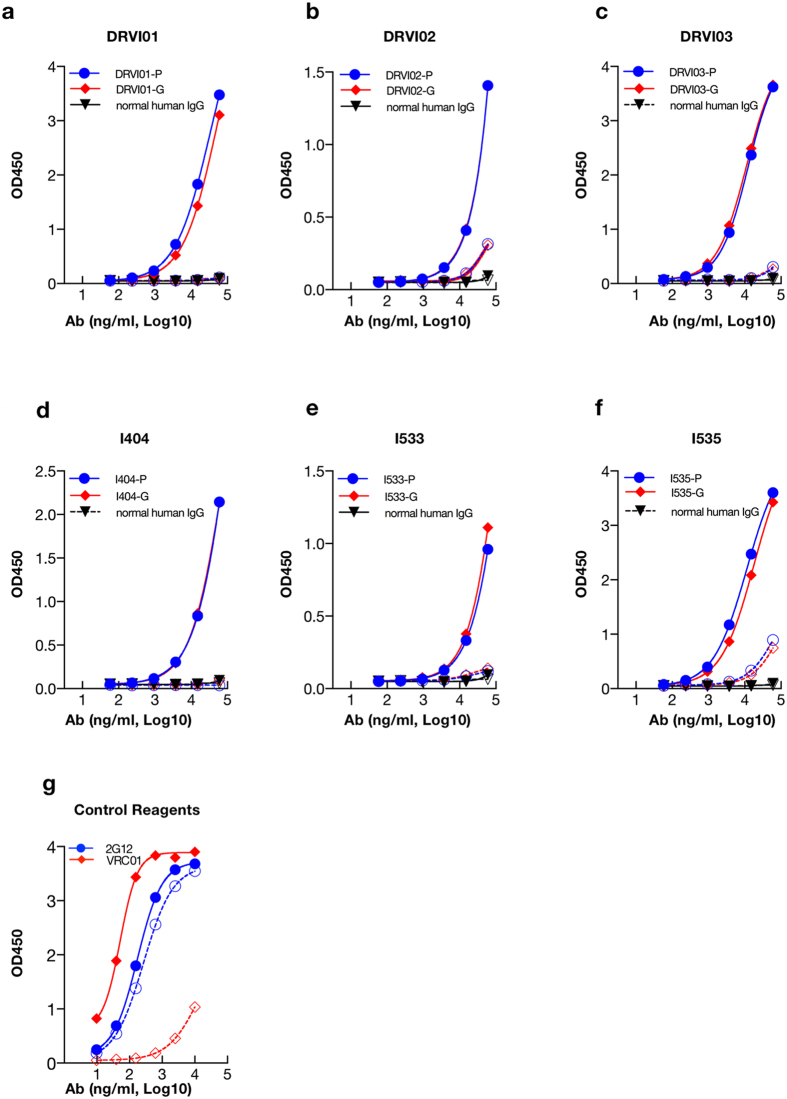
Binding profiles of six plasmas/IgGs screened from each cohort to RSC3/∆RSC3. Antigen binding profiles of six plasma samples and corresponding purified total IgGs ((**a**) DRVI01; (**b**) DRVI02; (**c**) DRVI03; (**d**) I404; (**e**) I533; (**f** ) I535)) including ((**g**) VRC01 and 2G12) were determined by ELISA assay. Solid symbols indicate plasma, IgG or mAbs binding to RSC3. Open symbols exhibit plasma, IgG and mAbs binding to ∆RSC3. Abbreviation of P indicates plasma sample and G represents IgG fraction.

The CD4 binding site (CD4bs), the initial site of gp120 attachment to the cellular receptor CD4^8^, represents one of the conserved neutralization epitopes on the HIV-1 Env trimers. Recently, researchers developed a pairing of HIV-1 core Env glycoproteins-antigenically resurfaced stabilized core 3 (RSC3) specific for the structurally conserved site of CD4 receptor binding and its mutant (∆RSC3, which lacked a single amino acid at position 371 that eliminated b12 binding, used as negative control)^34^. To investigate whether CD4bs-directed specificity existed in our top elite neutralizers, we utilized this pairing of Env probes to interrogate those samples and their corresponding IgG fractions. Six plasma samples (DRVI01, DRVI02, DRVI03 from FPD cohort and I404, I533, I535 from IDU cohort in Fig. 4a–c and d–f, respectively) possessed greater antigen binding activity to RSC3 than ∆RSC3, indicating the presence of CD4bs-directed antibodies in these six individuals (Fig. 4). Purified IgG shared the same binding profiles with the corresponding unfractionated plasma sample, suggesting that binding activity to RSC3 was mediated by the IgG fraction of the plasma.

### Heat-map analysis based on K-means clustering on neutralization data in IDU cohort

**Figure 5:**
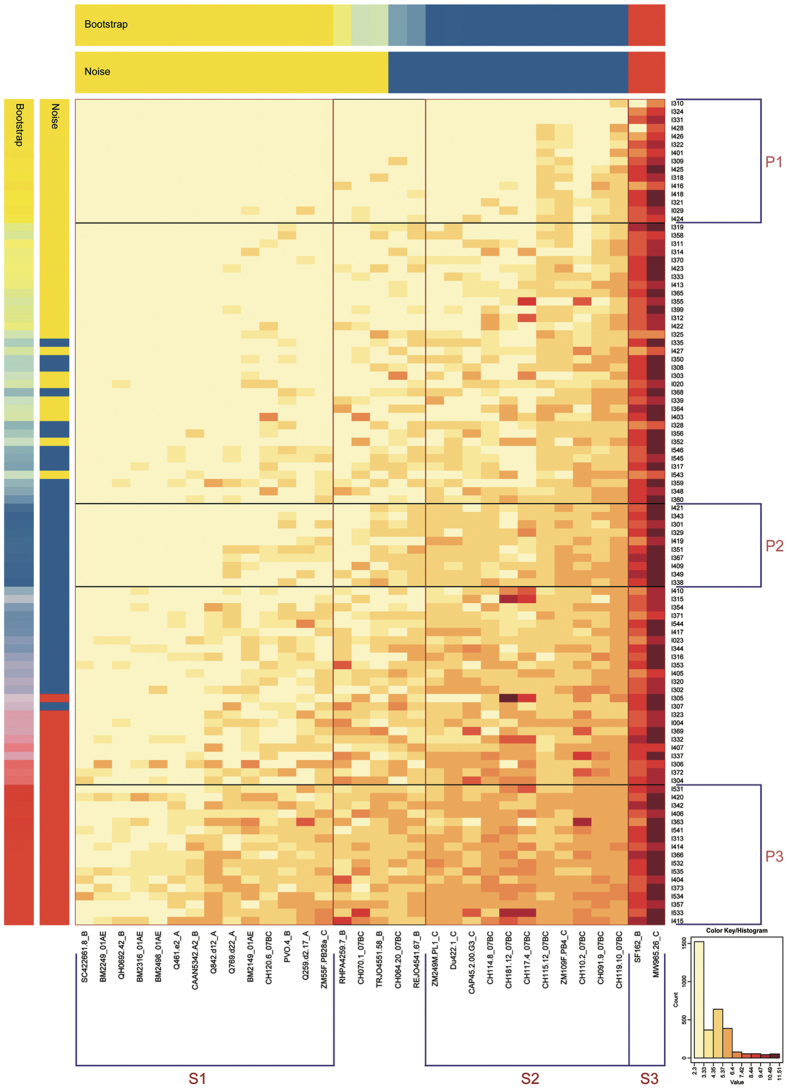
Heat-map analysis of all plasma samples based on k-means clustering. Transformed natural log data of ID_50_ values for 98 plasma samples against 32 virus strains are illustrated. In the heat-map, natural log ID_50_ values for a single plasma are shown by row, while virus strains are displayed by column. Stronger neutralization is represented by darker colors (see key based on log-transformed values). Vertically, the order of the plasma is ranked based on the GMT; the placement of clusters within this ranking is based on the mean titer for all cluster members. Bars with the label “Bootstrap” or “Noise” show the results of statistical analysis of clustering. Both are indicated by mixing the red, yellow and blue colors corresponding to the relative frequencies of matched group assignments. If they have a categorization of 90% or greater consistency, plasma and virus strains are grouped by both the bootstrap and noise tests. Boxes highlight the clusters. Clusters of patient plasma are labeled P1, P2, and P3 (from top to bottom), while clusters of strains are labeled S1, S2, and S3 (from left to right).

We probed for patterns of NAb responses in IDU cohort and underlying similarities in neutralization pattern utilizing the web-based heat-map tool. After k-means clustering (k = 3) and synchronous treatment of two statistical indices (“Bootstrap” and “Noise”), we identified three robust subgroups for both plasma and virus strains (Fig. 5).

The panel of diverse viral strains used to assess neutralization breadth could be grouped into three clusters (Fig. 5). Strain cluster 3 (S3) consisted of two tier 1 Env clones, MW965.26, and SF162.LS, the most neutralization-sensitive viruses in the panel^15^,^29^. Strains of different subtypes were included in two other clusters. Cluster S2 was exclusively composed of seven CRF07_BC envelopes and four subtype C envelopes. The most resistant cluster was S1, which included four subtype A, four CRF01_AE, four subtype B, one subtype C (ZM55F.PB28a), and one CRF07_BC strain (CH120.6), which was the most resistant tier 3 CRF07_BC virus strain^29^.

A considerable proportion of plasma samples characterized by the lack of neutralization breadth and potency were found to form plasma cluster P1 (Fig. 5). Plasma cluster P3 was composed of 17 plasma samples with the greatest neutralization breadth and potency, all of which were included in BCN group based on breadth classification, confirming the rationale of our breadth classification. Among the plasma samples with intermediate reactivity, one robust cluster was identified; plasma in this cluster (P2) did not neutralize the most resistant strains (GMT of 14.1 for P2 plasma versus S1 strains), but neutralized strains in the other two clusters with higher titers (GMT of 20417.9 and 131.9 for P2 plasma versus S3 and S2, respectively). By employing this approach, plasma clusters could be identified by overall breadth or potency and viral strain types that they neutralized.

## Discussion

We delineated the NAb profiles of CRF07_BC chronically infected individuals from an IDU cohort. We also compared NAb profiles between the IDU and FPD cohorts. Overall, data from both cohorts showed that more than 50% of plasma samples could neutralize at least 50% of virus strains tested, indicating that the human immune system is capable of mounting moderate neutralizing antibody responses in general, as has been observed previously in another large cohort study^31^. Therefore, it should be possible to design immunogens and optimize immunization strategies to induce similar NAb responses through vaccination.

Significantly higher NAb titers against intra-subtype viruses compared to inter-subtype virus strains were found in both cohorts, demonstrating the preferential intra-subtype neutralization independent of infection time and infecting virus subtype. Similar findings were also found in at least two reports, which showed the intra-subtype neutralization advantage^32^,^35^. One study demonstrated the subtype B-specific neutralization advantage in the subtype B infected Amsterdam cohort^32^ using multiple-subtype virus panel. The other study indicated this preferential neutralization effects in multiple subtype/CRF (subtype A, B, C, D, G, CRF01_AE, CRF02_AG, CRF07_BC, etc.) infected patients from different geographical regions including South Africa, America, Europe, and Asia^35^, etc. using large panels of genetically and geographically diverse HIV-1 Env-pseudotyped viruses. Taken together, these data suggest that subtype-specific immunogen design might be feasible to induce subtype-matched NAb responses in the vaccine context in various parts of the world, including in China. To date, the new generation Env trimers-the native-like trimer BG505 SOSIP.664 (subtype A) and B41 SOSIP.664 (subtype B)^36^-designed by utilizing structure biology together with serology knowledge elicited autologous NAb responses against resistant (tier 2) virus in various animal models including rabbits and rhesus macaques. The germline-targeting immunogens such as eOD-GT8^37^ and optimized 426c Env mutants (subtype C)^38^ triggered VRC01-class bNAb germline precursor B cells to initiate VRC01-class bNAb induction, supporting the use of these immunogens as a candidate prime vaccine. B cell lineage immunogen design^39^ concept offered an alternative approach to developing NAb responses targeting conserved CD4bs neutralizing antibody epitope. The optimal combination of these viable approaches using Env mutants derived from CRF07_BC may work best in the unique population to induce NAb responses, and this kind of research are actively being explored in this HIV-1 epidemic area. Furthermore, optimal coverage of mainly circulating subtype Env immunogens in given multiple subtypes-affected regions could combine the advantages of each subtype-specific immune response and maximize the potency and breadth coverage.

In addition, individuals chronically infected with CRF07_BC generated higher neutralization titers against the intra-subtype virus sets than those in subtype B’ subjects with a longer infection length, while subtype B’ subjects mounted broader and more potent neutralization responses against inter-subtype virus sets than those in CRF07_BC infections with shorter infection time, implying transition from narrow autologous to broad and potent heterologous neutralization over time. Previous studies of the molecular epidemiology for both cohorts demonstrated subtype B’^40^, and CRF07_BC^16^,^21^,^23^ shared similar virological features such as narrow ancestor virus and limited virus diversity, which could rule out virus diversity as a factor that influenced the NAb profile difference between these cohorts. Comparison of neutralization patterns between both cohorts with different infection time suggests that infection length might be one of the important factors for neutralization breadth development^41^,^42^. Initial autologous neutralization response targeting subtype-matched or intra-subtype viruses was very robust (Fig. 2a) and then shifted to inter-subtype strains (Fig. 3a) and became broader. Thorough understanding of this transition process with longitudinal follow-up study from acutely infected individuals will provide useful clues to vaccine immunogen design^43^.

A significant correlation between the neutralization potency and breadth was observed in both cohorts, which was in agreement with results from other American and European cohorts^31^,^32^. The dynamics of plasma neutralization activity in three elite neutralizers (Supplementary Fig. S5a: DRVI01 showed a gradual parallel increase in NAb potency and breadth over time during the observed periods, Supplementary Fig. S5b: DRVI02 exhibited a fluctuant evolution trend of NAb potency and breadth over time, Supplementary Fig. S5c DRVI03 displayed stable NAb potency and breadth over time) showed various patterns from the FPD cohort using multiple time points samples. These data also demonstrated NAb breadth and potency shared the same evolution trend during the study period (Figure S5) in a single elite neutralizer, which is in agreement with the trend of the overall study population in both cohorts (Figure S2)^15^. Neutralization breadth acquisition is vital for vaccine development since it can bypass immune escape exerted by continuous envelope variants. We found that GMTs against subtype-matched CRF07_BC viruses in the IDU cohort and GMTs against overall virus set in the FPD cohort correlated with viral load, suggesting that persistently evolving antigen exposure stimulated B cells to produce NAb with extensive affinity maturation^34^,^43^. This data sheds light on the necessity of optimizing immunization strategy, to include replication-competent vaccine vector utilization^44^,^45^, heterologous vector prime-boost regimen^46^ and modulation of antibody response using adjuvants^47^ to increase antigen loads and slowly release antigens in a long-term manner to repeatedly stimulate immune response maturation.

We demonstrated that plasma neutralization activity of the top six neutralizers from each cohort was attributable to the IgG fraction. Six elite neutralizers were able to mount strong RSC3-reactive CD4bs antibody. These CD4bs antibodies, detected in unfractionated plasma samples, were further confirmed to be mediated by IgG fraction. These data provided essential details about binding antibody specificity in the plasma and the antibody isotype (IgG dominantly), which can guide further isolation and identification of broadly neutralizing monoclonal antibodies to facilitate the NAb-based preventive and therapeutic strategies development^11^,^12^,^14^ in China and east Asia.

We identified a statistically robust cluster of plasma samples with broadly and potent NAb activity. These samples may provide us the opportunity to explore virological and immunological co-evolution patterns between pathogen and host, revealing the steps of bNAb induction in HIV-1 infected individuals. Recently, researchers tracked the development of a CD4bs bNAb lineage from one African donor^39^. Investigation of B cell responses in this donor showed cooperation between helper B cell lineage and broadly neutralizing B cell lineage to drive CD4bs bNAb development^48^. These critical studies imply that optimized Env immunogen design and immunization strategy such as sequential vaccination^49^ that could simultaneously stimulate both B cell lineages through immunization might recapitulate similar bNAb development process during natural infection.

In summary, we obtained the frequency, breadth, and potency of NAb responses in CRF07_BC infected individuals. Top elite neutralizers and the statistically robust cluster of plasma samples with broad or moderate NAb activity identified in the study are of particular interest for further monoclonal neutralizing antibody isolation and epitope specificity mapping^50^,^51^,^52^. An extensive study of the interaction between pathogen and host for elite neutralizers with exceptional broad and potent NAb responses might provide helpful information to guide regional NAb-based biomedical preventive and therapeutic approaches.

## Methods

### Study subjects

The study population consisted of 114 chronically HIV-1 infected individuals naïve to antiretroviral therapy (ART) at the time of sampling. After excluding 16 SVA-MLV (simian virus amphotropic murine leukemia virus) positive samples (ID_50_ > 20), the remaining 98 subjects were analyzed in this study. The study subjects consisted of 81 males and 17 females, with an average age of 32.7 ± 5.7 years old. Median CD4 counts and plasma viral loads at the time of sampling were 378 cells/mm^3^ (full range: 50–940 cells/mm^3^) and 20,700 copies/ml (full range: <50–714,000 copies/ml), respectively (Table S1). Subjects became infected through sharing contaminated needles during drug use, and subjects were confirmed to be infected with CRF07_BC strain^16^,^25^,^26^ (Table S1). The historical neutralization data of 103 subjects from the FPD cohort^15^ with over ten years’ infection time were used to compare with those generated in the IDU cohort. For all comparison involved in inter-subtype, the virus panel was restricted to the only matched viruses for both cohorts (Table S4). Upon the first detection of CD4 counts below 200, participants were encouraged to initiate free antiretroviral therapy according to the WHO and China government guidance for care and support for HIV/AIDS. This study was reviewed and approved by Institutional Review Board of the National Center for AIDS/STD Control and Prevention, Chinese Center for Disease Control and Prevention and all experiments in the current report were conducted in accordance with the relevant guidelines and regulation. All subjects provided written informed consent before blood (EDTA-anticoagulant used) and data collection.

### Cells

TZM-bl cells were obtained from the NIH AIDS Research and Reference Reagent Program (ARRRP, catalog no. 8129) as contributed by John Kappes and Xiaoyun Wu. 293T/17 cells were obtained from the American Type Culture Collection (catalog no. 11268). Both cell lines were maintained in Dulbecco’s modified Eagle’s medium (HyClone) containing 10% heat-inactivated fetal bovine serum (Hyclone) and 50 μg gentamicin/ml (Sigma) in vented T-75 culture flasks (Corning-Costar). The FreeStyle™ 293-F cell line was purchased from Invitrogen and was adapted to suspension culture in FreeStyle™ 293 Expression Medium. Cultures were incubated at 37 °C in a humidified 5% CO_2_–95% air environment with shaking at 225 rpm.

### Env-pseudotyped viruses

Molecularly cloned full-length gp160 were used for HIV-1 Env pseudovirus production by co-transfecting 293T/17 cells with an Env-deficient HIV-1 backbone plasmid (pSG3ΔEnv) as described^15^. The panel of 32 Env-pseudotyped viruses included ten CRF07_BC strains (CH064.2, CH070.1, CH091.9, CH110.2, CH114.8, CH115.1, CH117.4, CH119.1, CH120.6, CH181.12), five subtype C strains (Du422.1, ZM249M.PL1, ZM55F.P, ZM109F.PB4 and CAP45.2.00.G3), seven B strains (QH0692.42, SC422661.8, PVO.4, RHPA4259.7, REJO4541.67, TRJO4551.58, and CAAN5342.A2), four A strains (Q461.e2, Q769.d22, Q259.d2.17, Q842.d12), four CRF01_AE strains (BM2149, BM2249, BM2316 and BM2498), and two tier 1 viruses (MW965.26 for subtype C and SF162.LS for subtype B) (Table 1). HIV-1 Env pseudovirus stocks were titrated in TZM-bl cells described in our previous study^15^.

### NAb assay in TZM-bl cells

Plasma samples from patients and normal human plasma (HIV-1 negative) were heat inactivated at 56 °C for 30 min prior to assay. Neutralization was measured by using Tat-induced luciferase reporter gene expression to quantify reductions in virus infection in TZM-bl cells^15^,^53^,^54^. Briefly, 50 μl of the Env-pseudotyped virus was incubated with serial 3-fold dilutions of plasma samples/IgG fraction in a total volume of 150 μl for 1 hr at 37 °C in 96-well flat-bottom culture plates. Freshly trypsinized TZM-bl cells were then added (1 × 10^4^/well in a 100 μl volume) in 10% DMEM growth medium containing DEAE-dextran (Sigma, St. Louis, MO) at a final concentration of 10 μg/ml. One set of control wells received cells + pseudovirus (virus control) while another set received cells only (background control). Following 48 hrs incubation, 150 μl of culture medium was removed from each well, and 100 μl of a luciferase reporter gene assay system reagent (Bright-Glo, Promega) was added. After a short incubation (minimum of 2 minutes), 150 μl of lysate from each well was transferred to 96-well black solid plates (PerkinElmer Life Sciences) for measurement of luminescence in a luminometer (PerkinElmer Life Sciences, Victor X3). The ID_50_ or 50% inhibitory concentration (IC_50_) was defined as the reciprocal of the plasma reagent dilution or concentration of IgG fraction that caused a 50% reduction in relative luminescence units (RLU) compared to virus control wells after subtraction of background RLU. All NAb assays were conducted using the validated TZM-bl NAb assay^55^.

### Standard enzyme-linked immunosorbent assay (ELISA)

RSC3/ΔRSC3 proteins dissolved at a concentration of 1 μg/ml in phosphate-buffered saline (PBS, pH 7.4) were used to coat in 96-well ELISA plate at 100 μl/well overnight at 4 °C. Coated plates were blocked with blocking buffer (PBS, pH 7.4, plus 2% BSA and 5% milk) at 200 μl/well for 1 hr at room temperature, followed by incubation with plasma or IgGs/antibody serially diluted in PBST buffer (0.05% Tween 20 in PBS) at 100 μl/well for 1 hour at room temperature. Horseradish peroxidase (HRP)-conjugated goat anti-human IgG Fc antibody (Jackson ImmunoResearch Laboratories Inc., West Grove, PA) at 1:10,000 was added for 1 hr at room temperature at 100 μl/well. Plates were washed between each step with PBST buffer at 100 μl/well. Plates were developed using 100 μl of the substrate (KPL SureBlue TMB 1-Component Microwell Peroxidase Substrate, cat#: 52-00-02) and stopped with 50 μl 1 N H_2_SO_4_. Absorption at 450 nm was read on an automated plate reader (Multiscan Ascent, Thermo Corporation, Finland).

### Plasma IgG extraction and quantitative determination

Polyclonal IgG was purified from the plasma by using protein A affinity chromatography (GE Healthcare) according to the manufacturer’s instructions. IgG was eluted from the columns using 0.1 M citric acid, pH 3.0. Fractions containing IgG were neutralized, pooled, and dialyzed against PBS, pH 7.4. IgG in plasma and the purified preparation was quantified using a human IgG ELISA according to the instruction (Zeptometrix). A standard curve was plotted with serial dilutions of human IgG using CurveExpert 1.4 curve fitting software. Both the plasma and IgG were then tested at starting concentrations equivalent to 200 μg/ml on the selected virus panel (Table 2) for the NAb assay (initial concentration is 4 mg/ml) or 60 μg/ml to do ELISA test (initial concentration is 6 mg/ml).

### Viral load testing

Plasma viral load was measured using the Amplicor Ultrasensitive assay (Hoffman-La Roche) according to the manufacturer’s instructions. This assay has a lower detection limit of 50 copies HIV-1 RNA ml^−1^.

### CD4^+^ T cell count

CD4^+^ T cell counts from EDTA-anticoagulated whole blood were assessed using TruCounts (FITC-CD3Ab, PE-CD4Ab, PerCP-CD45Ab, APC-CD8Ab) and FACSCalibur (BD).

### Heatmap analysis based on kmeans (k = 3) clustering

We utilized the heatmap tool based on kmeans clustering (k = 3), as publically available on the Los Alamos HIV database website (http://www.hiv.lanl.gov/content/sequence/HEATMAP_KMEANS/heatmap_kmeans.html). This web-based heatmap tool used is a modified version of “heatmap.2” of the gplots package of the statistical environment R^56^. Bootstrap was used to evaluate the stability of components in a certain subgroup by resampling with replacement 10,000 times. Similarly, noise data representing the repeated iterations data were used to evaluate the impact of assay-to-assay variability. The resulting degree of consensus is shown in the row or column labeled “Bootstrap” in Fig. 5. Stability of categories for these data is shown in the row and column labeled “Noise” in Fig. 5. We defined three subgroups (k = 3) as the maximum number such that three robust clusters representing higher, moderate and lower levels for both plasma and virus strains were formed at a consistency of at least 90% by both indices of stability. These methods were also described previously^15^,^29^.

### Statistical analyses

Statistical analysis and basic graphical delineation were performed using GraphPad Prism 7 (GraphPad Software Inc.) and Microsoft Excel 2011 (Microsoft Corp.) based on a Spearman rank correlation or a Mann-Whitney U test, where appropriate; *P* < 0.05 was considered significant. Viral load values below the limit of detection (50 RNA copies/ml) were assigned a value of 49 for statistical analysis purposes.

## Additional Information

**How to cite this article:** Hu, X. *et al*. Profiling the neutralizing antibody response in chronically HIV-1 CRF07_BC-infected intravenous drug users naïve to antiretroviral therapy. *Sci. Rep.* **7**, 46308; doi: 10.1038/srep46308 (2017).

**Publisher's note:** Springer Nature remains neutral with regard to jurisdictional claims in published maps and institutional affiliations.

## Supplementary Material

Supplementary Information

## Figures and Tables

**Table 1:** Neutralization magnitude distribution between different sample groups based on neutralization breadth.Note: GMTs are shown. ID_50_ values < 20 (lowest sample dilution tested) were assigned a value of 10. Bold type indicates subtype-specific values.^*^indicates Tier 3 virus, ^†^indicates Tier 1B virus.

	Control	Tier 1 A (n = 2)	CRF07_BC (n = 10)												Subtype A (n = 4)				
Group based on NAb breadth	SVA-MLV	MW965.26	SF162.LS	CH064. 20	CH070.1^*^	CH091.9	CH110.2	CH114.8^*^	CH115. 12^*^	CH117.4	CH119. 10	CH120.6^*^	CH181. 12	CRF- specific	Q461.e2	Q769.d22	Q259.d2. 17	Q842. d12	Subtype- specific
**BCN (n = 18)**	10	56334	4901	184.3	160.3	470.5	518.0	361.2	250.5	503.1	520.4	89.3	630.2	**314.9**	39.1	91.7	170.2	156.5	**98.8**
**M-BCN (n = 34)**	10	42668	5166	64.6	38.9	241.1	289.0	140.0	111.3	142.1	340.2	26.1	93.1	**112.3**	13.0	33.1	32.2	24.9	**24.2**
**Non-BCN(n = 46)**	10	23864	2903	22.4	20.1	84.5	40.4	28.2	83.4	34.8	191.8	15.7	31.1	**40.5**	10.0	10.7	12.3	10.0	**10.7**
**Overall (n = 98)**	10	34183	3903	47.6	37.0	166.7	127.8	78.5	112.8	92.6	281.0	25.7	79.0	**84.1**	14.1	23.5	27.8	22.8	**21.4**
	**Subtype C (n = 5)**						**Subtype B (n = 7)**								**CRF01_AE (n = 4)**				
	**Du422.1**	**ZM249M.PL1**	**ZM55F.PB28a**	**ZM109F.PB4**^**†**^	**CAP45.2.00.G3**	**Subtype-specific**	**QH0692.42**	**SC422661.8**	PVO.4^*^	**RHPA4259.7**	**REJO4541.67**	TRJO4551.58^*^	**CAAN5342.A2**	**Subtype-specific**	**BM2149**	**BM2249**	**BM2316**	**BM2498**	**CRF-specific**
**BCN(n = 18)**	250.3	242.2	142.3	243.7	206.4	**212.6**	25.4	20.2	87.4	149.1	169.7	171.5	46.8	**71.0**	131.1	21.9	24.7	24.2	**36.2**
**M-BCN(n = 34)**	116.5	115.1	44.6	179.2	132.7	**107.3**	11.9	11.8	31.1	53.6	83.9	56.4	13.1	**28.3**	28.4	12.2	13.2	14.0	**15.9**
**Non-BCN(n = 46)**	22.9	22.7	11.9	70.2	23.7	**25.3**	10.0	10.0	14.6	14.4	22.0	17.6	10.9	**13.7**	12.3	10.0	10.0	10.0	**10.5**
**Overall (n = 98)**	62.5	61.6	29.6	122.1	64.2	**61.7**	12.6	12.1	26.3	34.9	51.0	40.1	15.2	**23.8**	25.3	12.4	13.0	13.2	**15.2**

**Table 2:** Spearman rank correlation of the association between plasma and IgG neutralization by Env-pseudotyped virus^a^.

Env-pseudotyped virus	R	R^2^	*P* value
SF162.LS	0.8455	0.715	0.001
CH110.2	0.9455	0.894	<0.0001
CH181.12	0.7909	0.626	0.0037
CH120.6	0.8333	0.694	0.0014
QH0692.42	0.6057	0.367	0.0483
PVO.4	0.8273	0.684	0.0017
REJO4541.67	0.8095	0.655	0.0025
Du422.1	0.8716	0.760	0.0005
ZM109F.PB4	0.8349	0.697	0.0014
Q769.d22	0.7982	0.637	0.0032
BM2316	0.7762	0.602	0.0050

^a^ID_50_ titers above the upper limit of detection were assigned a value of 200 units, equal to the upper limit of detection.
