# Supplementary material for: Profiling the neutralizing antibody response in chronically HIV-1 CRF07_BC-infected intravenous drug users naïve to antiretroviral therapy
Source: Sci Rep. 2017 Apr 7;7:46308. doi: 10.1038/srep46308 (PMC5384219; doi:10.1038/srep46308)
Supplement: Supplementary Information [file srep46308-s1.pdf]

Supplementary Information (SI) for

Profiling the neutralizing antibody response in chronically HIV-1 CRF07_BC-infected intravenous drug users naïve to antiretroviral therapy

Xintao Hu[1,§,†], Yuanyuan Hu[1,†], Chunhong Zhao[1], Hongmei Gao[2], Kelli Greene[2], Li Ren[1], Liying Ma[1], Yuhua Ruan[1], Marcella Sarzotti-Kelsoe[2,3], David C. Montefiori[2], Kunxue Hong[1,*], Yiming Shao[1,*]

[1]State Key Laboratory of Infectious Disease Prevention and Control, National Center for AIDS/STD Control and Prevention, Chinese Center for Disease Control and Prevention, Collaborative Innovation Center for Diagnosis and Treatment of Infectious Diseases，Beijing 102206, PR China.

[2]Departments of Experimental Surgery and [3]Immunology, Duke University Medical Center, Durham, North Carolina 27710, USA

[§]Current affiliation: Human Retrovirus Pathogenesis Section, Vaccine Branch, Center for Cancer Research, National Cancer Institute, Frederick, Maryland 21702, USA

† these authors contributed equally to this work

[*]**Corresponding author:** Kunxue Hong & Yiming Shao

Tel: 86-10-58900981; Fax: 86-10-58900980

Email: hongkx@chinaaids.cn; yshao08@gmail.com

This file includes:

Supplementary Tables S1-4; Supplementary Figures S1-5

Table S1: Major characteristics of IDU cohort.

Category	Site		
	**Xinjiang (n=66)**	**Sichuan (n=32)**	**Total (n=98)**
Age (years; mean±SD)	32.7±5.1	32.8±6.8	32.7±5.7
Gender [% (n)]			
Male	81.8(54)	84.4(27)	83(81)
Female	18.2 (12)	15.6(5)	17(17)
Ethnic Group [% (n)]			
Uygur	81.8(54)	0(0)	55(54)
Han	16.7(11)	78.1(25)	37(36)
Yi	0(0)	21.9(7)	7(7)
Hui	1.5(1)	0(0)	1(1)
High-Risk behavior [n (%)]			
Intravenous drug use	66(100.0)	32(100.0)	98(100.0)
Former plasma donation	0	0	0
HIV-1 subtype/CRF	CRF07_BC	CRF07_BC	CRF07_BC
CD4 count number (cells/mm^3; median)	341	404	378
(range)	(50-940)	(118-834)	(50-940)
Viral load (copies/ml; median)	27900	9930	20700
(range)	(<50-563000)	(<50-714000)	(<50-714000)
Infection Length (years)		3-5	

Table S2: Summary of top 6 neutralizers from both cohorts.

Patient ID (#code)	Site	Gender	Age	Ethnic	Infection via*	CD4 Count (cells/mm^3)	Plasma RNA Viral Load (copies/ml)	NAb breadth (%)	NAb potency (GMT)	Subtype
DRVI01	Anhui	M	44	Han	FPD	512	268000	96	302.2	B’
DRVI02	Anhui	M	48	Han	FPD	268	85600	96	256.6	B’
DRVI03	Anhui	M	35	Han	FPD	373	79800	100	185.7	B’
F438	Anhui	M	47	Han	FPD	480	93800	96	143.7	B’
F433	Anhui	M	39	Han	FPD	453	51500	91	102.8	B’
F521	Anhui	F	65	Han	FPD	997	202000	96	101	B’
I415	Sichuan	M	42	Han	IDU	310	6420	97	295.9	CRF07_BC
I533	Xinjiang	M	35	Wei	IDU	57[#]	236000	90	234.7	CRF07_BC
I534	Xinjiang	M	27	Wei	IDU	164[#]	277000	97	173.6	CRF07_BC
I404	Sichuan	M	25	Yi	IDU	542	116000	93	166.4	CRF07_BC
I357	Xinjiang	M	38	Wei	IDU	628	10500	83	164.9	CRF07_BC
I535	Xinjiang	M	32	Wei	IDU	336	144000	100	160.4	CRF07_BC

*FPD: former plasma donation; IDU: intravenous drug use

[#]demonstrating the participants who initiated free antiretroviral therapy upon first detection of CD4 counts below 200

Table S3: Concentration determined by quantitative ELISA for top 6 plasma samples in both cohorts.

FPD cohort		IDU cohort	
**Sample ID#**	**Concentration (μg/μl)**	**Sample ID#**	**Concentration (μg/μl)**
DRVI01-P[a]	29.2	I415-P[a]	15.2
DRVI01-G[b]	10.8	I415-G[b]	7.2
DRVI02-P[a]	24.2	I404-P[a]	14.8
DRVI02-G[b]	7.7	I404-G[b]	8.5
DRVI03-P[a]	18.3	I357-P[a]	15.7
DRVI03-G[b]	6.5	I357-G[b]	6.4
F438-P[a]	10.4	I533-P[a]	13.6
F438-G[b]	4.0	I533-G[b]	6.0
F433-P[a]	11.8	I534-P[a]	15.7
F433-G[b]	5.6	I534-G[b]	6.8
F521-P[a]	17.5	I535-P[a]	13.5
F521-G[b]	6.6	I535-G[b]	8.6

[a] P indicates plasma sample and [b] G represents for IgG fraction

Table S4: Matched virus panel for both cohorts used for inter-subtype comparison.

Subtype	Virus Strain	Subtype	Virus Strain
B (n=7)	QH0692.42	C (n=4)	Du422.1
	SC422661.8		ZM249M.PL1
	PVO.4[*]		ZM109F.PB4[†]
	RHPA4259.7		CAP45.2.00.G3
	REJO4541.67	A (n=4)	Q461.e2
	TRJO4551.58[*]		Q769.d22
	CAAN5342.A2		Q259.d2.17
CRF07_BC (n=4)	CH110.2		Q842.d12
	CH117.4	CRF01_AE (n=3)	BM2249
	CH120.6[*]		BM2316
	CH181.12		BM2498

Note: [*]indicates Tier 3 virus, [†]indicates Tier 1B virus

Supplementary Figure S1

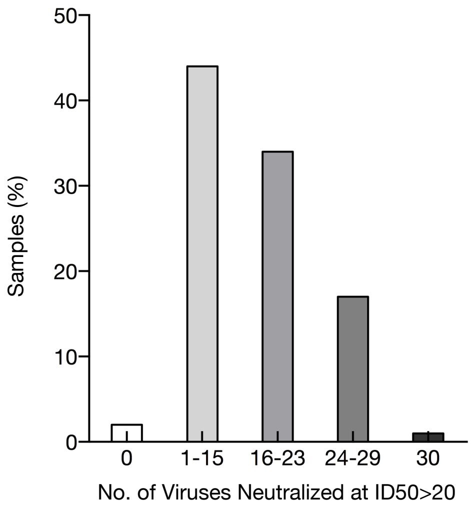

Figure S1: Distribution of neutralization breadth among the overall plasma sample population. Neutralization breadth is defined as the percentage of strains neutralized at a detectable titer (ID_{50}>20) by individual plasma sample out of the 30 strains tested (the two tier 1 viruses, MW965.26 and SF162.LS excluded). Each bar indicates the percentage of samples, out of 98 in total, neutralized by the corresponding number of viruses as indicated on the x-axis.

Supplementary Figure S2

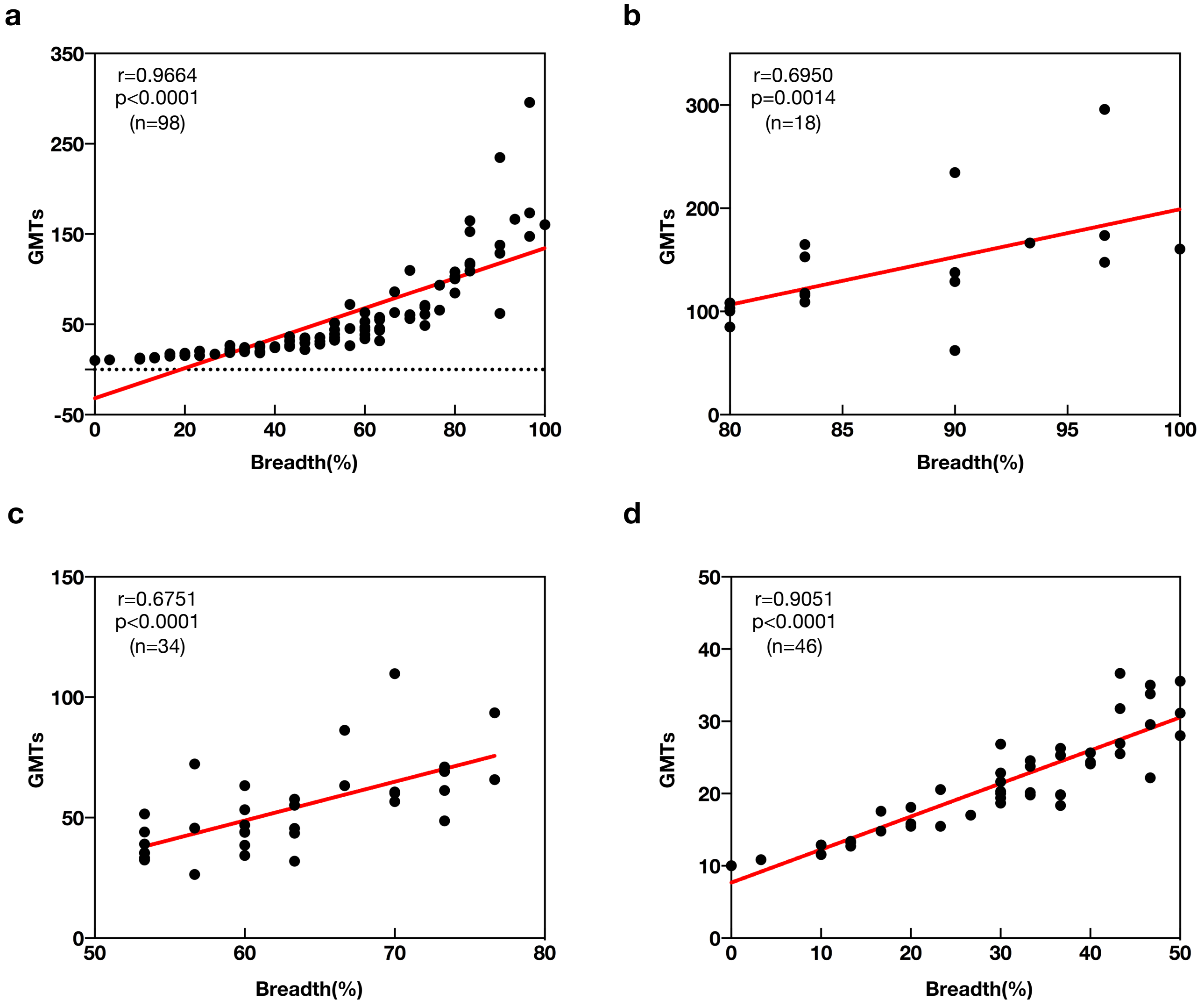

Figure S2: Correlation between NAb breadth and potency in IDU cohort.
For the different population (a: overall samples; b: BCN samples; c: M-BCN samples; D: non-BCN samples), the neutralization magnitudes are compared with neutralization breadth, respectively. P-values (two-tailed) and R-values are based on the Spearman's rank test.

Supplementary Figure S3

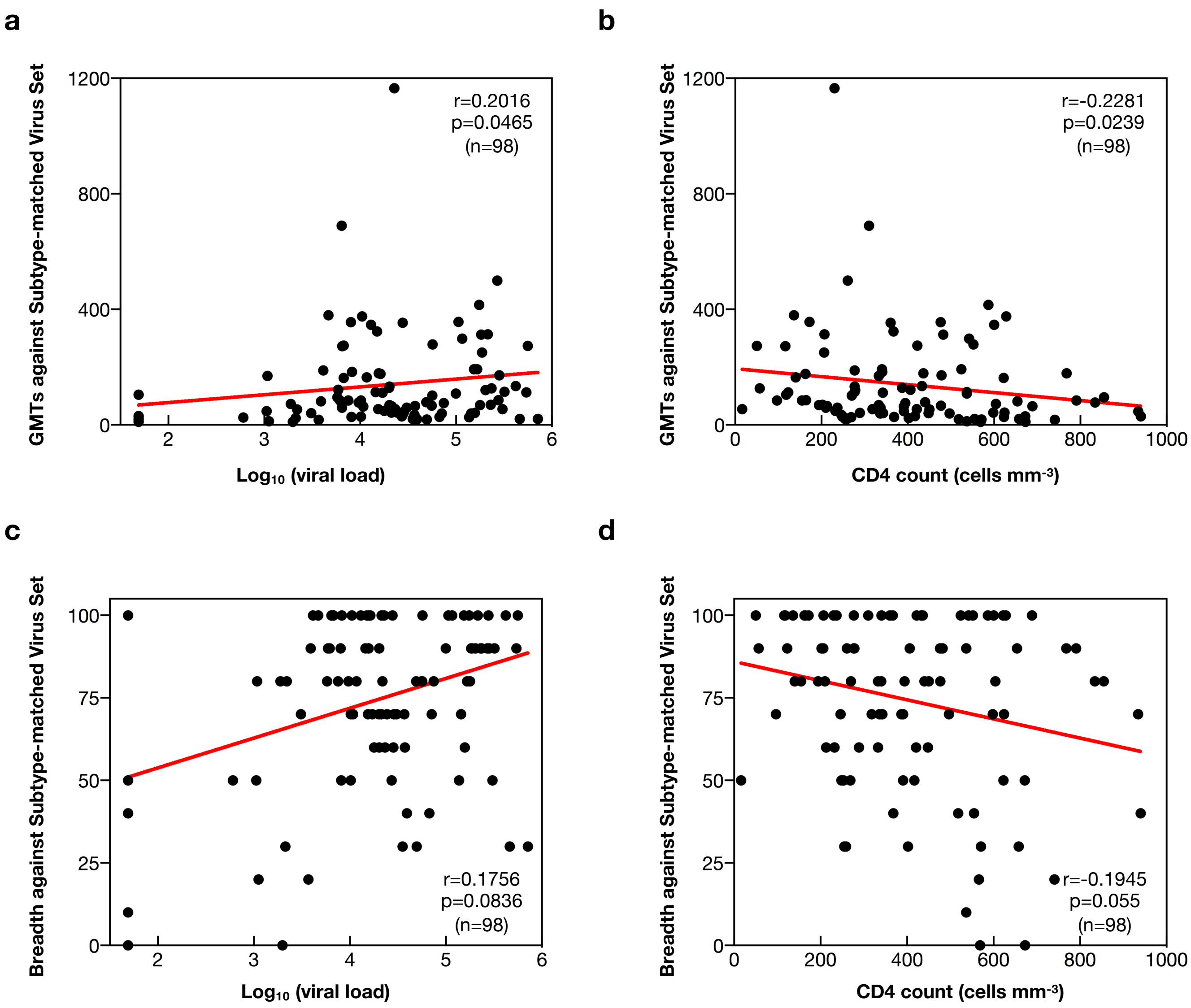

Figure S3: Correlation between neutralization magnitude against subtype-matched virus set and plasma viral loads/CD4 counts in IDU cohort.

For the total population of 98 samples, the neutralization magnitudes against subtype-matched virus set of NAb response are compared with individuals' viral load (a) and CD4 counts (b), respectively. Similarly, the neutralization breadth (%) against subtype-matched virus set of NAb response are compared with individuals' viral load (c) and CD4 counts (d), respectively. P-values (two-tailed) and R-values are based on the Spearman's rank test.

Supplementary Figure S4

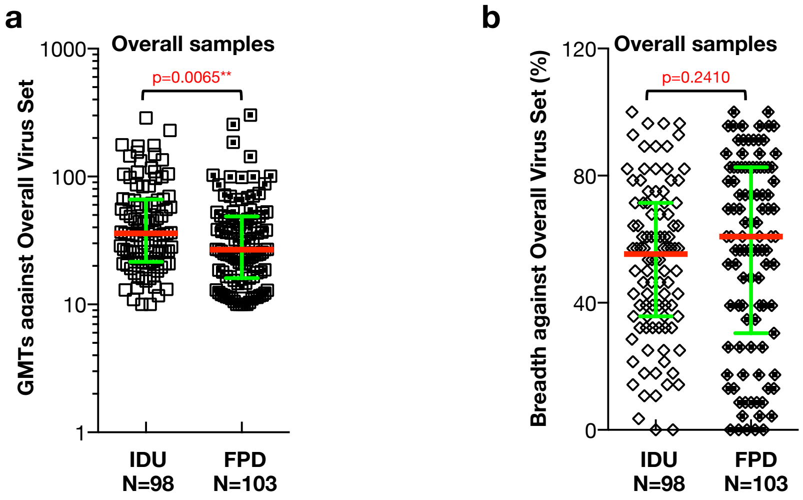

Figure S4: Comparison of NAb magnitude and breadth against overall virus set between FPD and IDU cohort.

The difference of NAb magnitude (a) or breadth (b) against overall virus set between IDU and FPD cohort is illustrated for the overall sample population. P-values (two-tailed) are based on the Mann-Whitney U test. The error bars show the median with the interquartile range. The Significant difference between each group is indicated: *0.01<P<0.05; **0.001<P<0.01; ***P<0.001 ; ****P<0.0001 (Mann-Whitney U test).

Supplementary Figure S5

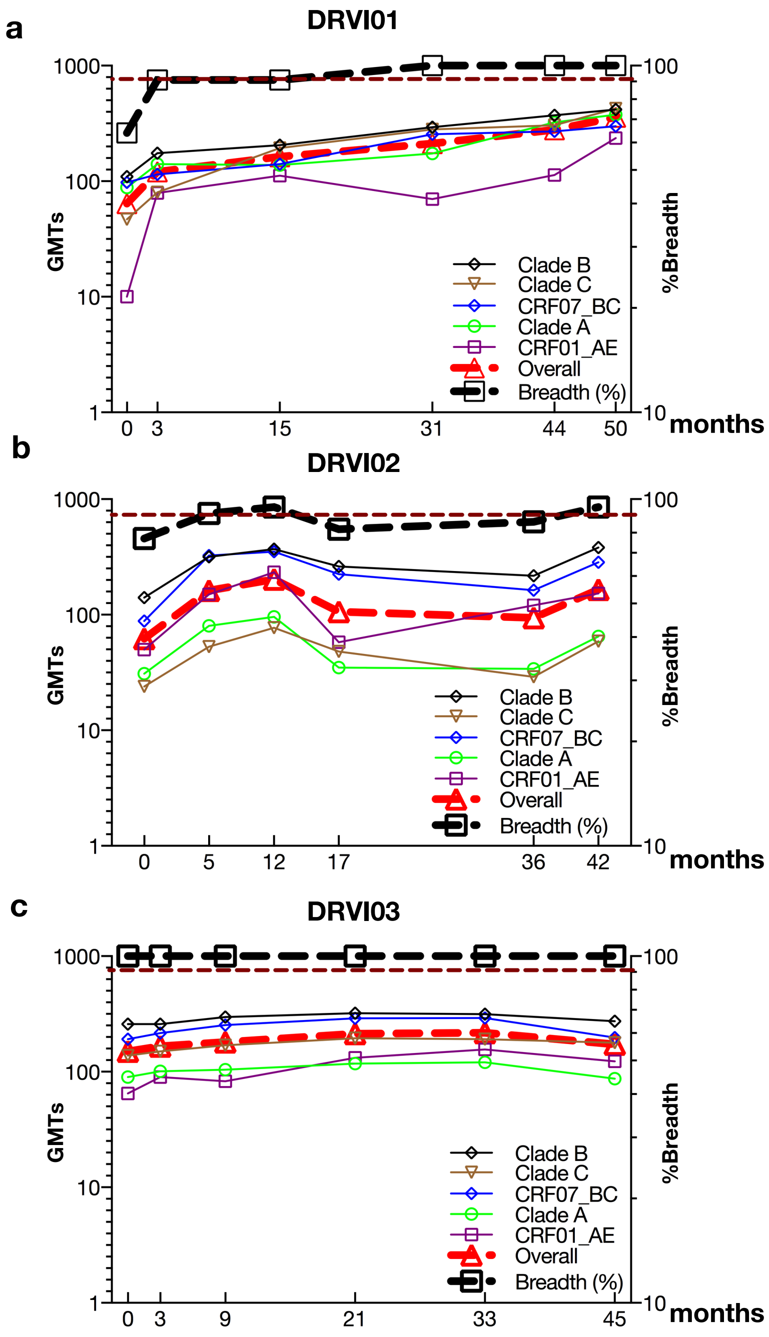

Figure S5: Dynamics of top 3 plasma neutralization activity evolution in FPD cohort.
Six time points (months) of top three plasma samples (DRVI01 (a), DRVI02 (b) and DRVI03 (c)) from FPD cohort are shown on the x-axis. The y-axis (left) values represent the GMTs of plasma neutralization against corresponding subtype-specific/overall virus set, and the y-axis (right) shows the neutralization breadth (%) against overall virus set. The dark red dotted line representing 90% breadth threshold was plotted in each sample's figure. Plasma samples with approximately 3-16, 5-19, and 3-12 month intervals for DRVI01, DRVI02, DRVI03, respectively, were analyzed.
